# Dog and mouse: toward a balanced view of the mammalian olfactory system

**DOI:** 10.3389/fnana.2014.00106

**Published:** 2014-09-25

**Authors:** Arthur W. Barrios, Pablo Sánchez-Quinteiro, Ignacio Salazar

**Affiliations:** Unit of Anatomy and Embryology, Department of Anatomy and Animal Production, Faculty of Veterinary, University of Santiago de CompostelaLugo, Spain

**Keywords:** olfactory epithelium, olfactory subsystems, morphology, evolution, dog

## Abstract

Although the most intensively studied mammalian olfactory system is that of the mouse, in which olfactory chemical cues of one kind or another are detected in four different nasal areas [the main olfactory epithelium (MOE), the septal organ (SO), Grüneberg's ganglion, and the sensory epithelium of the vomeronasal organ (VNO)], the extraordinarily sensitive olfactory system of the dog is also an important model that is increasingly used, for example in genomic studies of species evolution. Here we describe the topography and extent of the main olfactory and vomeronasal sensory epithelia of the dog, and we report finding no structures equivalent to the Grüneberg ganglion and SO of the mouse. Since we examined adults, newborns, and fetuses we conclude that these latter structures are absent in dogs, possibly as the result of regression or involution. The absence of a vomeronasal component based on VR2 receptors suggests that the VNO may be undergoing a similar involutionary process.

## Introduction

The dog is increasingly appreciated in biomedical research as a species that, unlike purpose-bred laboratory animals, shares the genetic, and clinical variety of human patients (Karlsson and Lindblad-Toh, 2008). Its nasal cavity has been studied from various points of view. Anatomically, Graeger's paper (Graeger, 1958) is considered the classical reference. Physiologically, attention has recently focused on nasal airflow patterns (Craven et al., 2007, 2010). Clinically, the presentation, diagnosis, prognosis, and treatment of tumors is a major current concern (McEntee, 2004; Turek and Lana, 2012; Mason et al., 2013), but the olfactory mucosa of the nasal cavity has also attracted clinical interest because of its unique maintenance of a population of basal cells supporting the continual regeneration of olfactory sensory neurons (OSNs) (Graziadei and Monti-Graziadei, 1979); the intraspinal implantation of cells derived from autologous olfactory mucosa cultures has in fact recently been successful in ameliorating the effects of spinal cord injuries in companion dogs (Granger et al., 2012).

In the mouse, the animal in which the mammalian olfactory system has been most intensively studied, the nasal mucosa features four separate olfactory areas: the main olfactory epithelium (MOE), the septal organ (SO), the ganglion of Grüneberg (GG), and the vomeronasal sensory epithelium [VNsE, not to be confused with the vomeronasal organ (VNO) of which it forms a part] (Breer et al., 2006; Munger et al., 2009; Ma, 2010; Barrios et al., 2014). These four sensory areas can be considered as the points of entry to four olfactory subsystems (OSbS), the integration of which at higher levels is a focus of current research. However, this four-subsystem scheme is by no means exhibited by all mammals, or even by all macrosmatic mammals (Salazar and Sánchez-Quinteiro, 2009). In the work described here we examined its validity for the dog, a notoriously macrosmatic animal. We found that the dog has no GG or SO, and that its VNO shows signs of similar involution.

## Materials and methods

### Animals

The dogs studied were 31 male or female mesaticephalic adults, 17 newborn males or females from four different litters, and 16 fetuses obtained on days 30, 35, or 40 of gestation. Most were German Shepherds or mongrels derived therefrom, and all were mesaticephalic. All were obtained legally through the dissecting and post-mortem rooms and Department of Clinical Science of our faculty and were treated in accordance with Spanish and EU legislation for the care and handling of animals in research (RD 223/1998, 86/609/EEC) and with the guidelines of the University of Santiago de Compostela Bioethical Committee. The heads of all animals were intact and showed no clinical or post-mortem evidence of neurological disease; all were processed as specified below as soon after death as was possible.

### Processing of samples and tissue sections

Using traditional anatomical techniques, 22 adult heads were carefully prepared by dissection and micro-dissection—from outside to inside—to afford views of the lateral and medial walls of the nasal cavity and lateral and medial views of the turbinates. Views were systematically recorded in photographs and drawings, and the chromatic characteristics of the mucosa were noted. The separate components of the turbinate complex were then dissected and prepared.

Seven puppy heads were similarly prepared except for the final dissection of the turbinate complex.

Transverse sections of uniform thickness were cut on a polystyrene block from two adult heads that had been washed and frozen following appropriate cleaning, fixation by immersion in 10% formaldehyde for 96 h, and removal of the mandibles and associated structures.

Ten puppy heads were prepared for histological examination as follows. Eight heads (group 1) were fixed by immersion in neutral buffered formaldehyde, where they remained until use, and two (group 2) were immersed in Bouin's fixative for 24 h and then transferred to 70% alcohol. For examination of the whole nasal cavity (mainly with a view to delimiting the MOE), two group 1 heads were decalcified in Shandon TBD-1 rapid decalcifier (Thermo, Pittsburgh, PA) and embedded in one piece, appropriately oriented, in paraffin wax, after which serial transverse sections 8–10 μm thick were cut. 710 alternate sections from one of these heads, and 710 corresponding sections from the other, were transferred to slides and stained with hematoxylin-eosin (HE). The remaining eight heads (six from group 1 and the two group 2 heads) were prepared, sectioned and stained in the same way, except that before sectioning the nasal cavity was divided transversally into three blocks of more or less equal length.

All fetal heads were processed for histological examination in the same way as the puppy heads, with the main goal of scrutinizing the regions in which the GG and SO were expected to be found, if present. The posterior nasal cavity was also studied.

Owing to the difficulty of histological preparation of the whole adult nasal cavity, the following subregions of the remaining seven adult heads were excised, decalcified, and embedded in paraffin wax for sectioning and subsequent staining of the sections with HE or antibodies (see below): (i) four levels of the nasal septum; and (ii) the individual turbinates.

### Immunohistochemistry

Immunohistochemical studies were performed using antibodies against olfactory marker protein (OMP) (Wako Chemicals, 1:500 dilution) and the G-proteins G_αi_2 (Santa Cruz Biotechnology, 1:100) and G_αo_ (Santa Cruz Biotechnology and Medical & Biological Lab Co., 1:100). Sections were dewaxed in xylene, rehydrated, and successively incubated (1) for 30 min at room temperature in PBS containing 5% normal horse serum and 2% bovine serum albumin, (2) for 24 h at 4°C in primary antibody solution, (3) for 1 h in biotinylated secondary antibody solution, and (4) for 2 h in a solution of avidin-biotin-horseradish peroxidase complex (ABC Vectastain reagent); after which standard procedures for visualization of the horseradish peroxidase complex with 3,3-diaminobenzidine were followed, and the sections were dehydrated through alcohols, cleared in xylene, and coverslipped. Sections of formalin- or Bouin-fixed canine and murine olfactory bulbs were used as control tissues.

### Image acquisition and processing

Digital images were captured using a Karl Zeiss Axiocam MRc5 digital camera. When necessary, Adobe Photoshop 6.0 (Adobe Systems, San Jose, CA) was used to adjust contrast and brightness to equilibrate light levels, and/or to crop, resize, and rotate the images for presentation; no additional digital image manipulation was performed.

## Results

**Figure 1:**
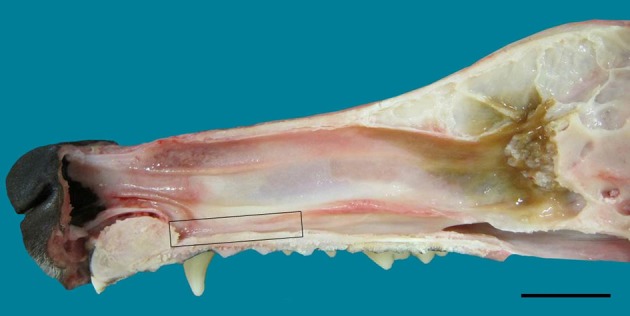
**Lateral view of the nasal septum of an adult dog, showing the difference in color between sensory (yellow-brown) and respiratory (red-orange) mucosa**. The rectangle frames the vomeronarasal organ. Scale bar: 2 cm (see Figure S1).

**Figure 2:**
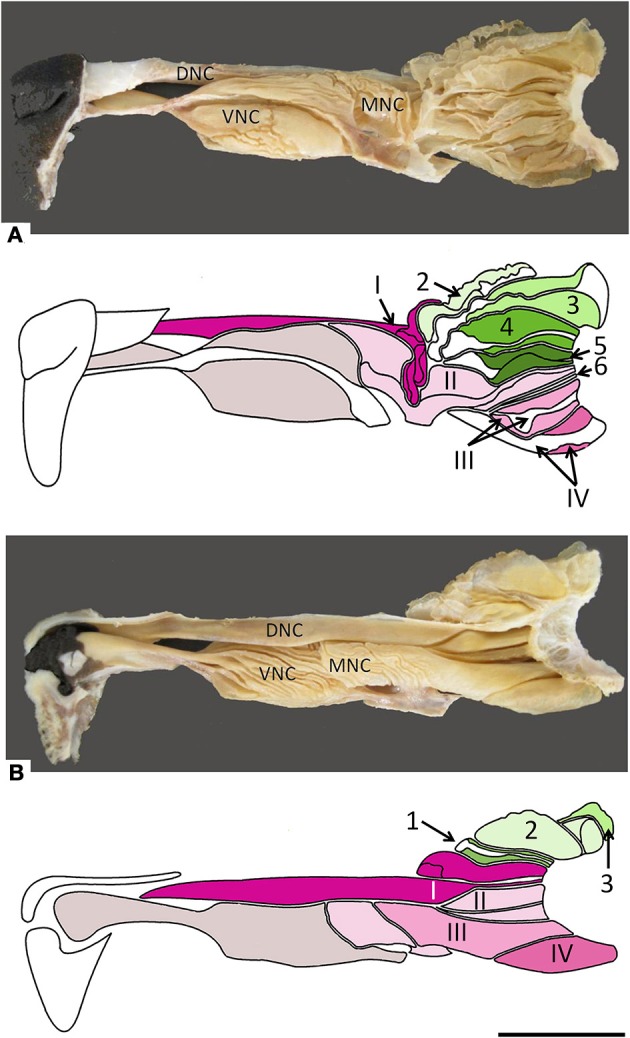
**Photographs and schematic drawings of the turbinate complex in lateral (A) and medial (B) views**. DNC, dorsal nasal concha; MNC, middle nasal concha; VNC, ventral nasal concha. Ectoturbinates are identified in the drawings by Arabic numerals (1–6) and shades of green, endoturbinates by roman numerals (I–IV) and shades of pink. Scale bar: 2 cm (see Figure S2).

**Figure 3:**
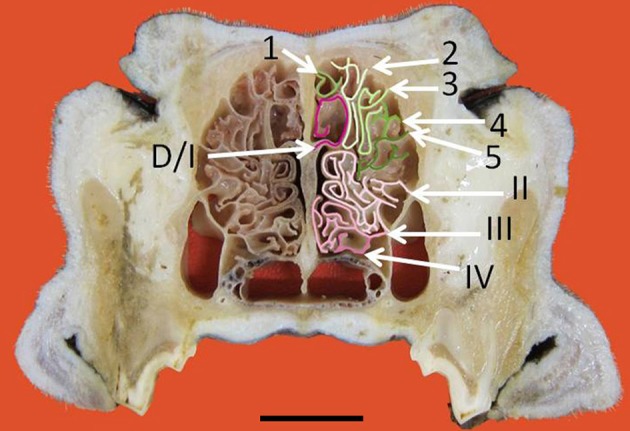
**Transverse frozen section of the nasal cavity**. D, dorsal nasal concha. Ectoturbinates are identified by Arabic numerals (1–5) and endoturbinates by roman numerals (I–IV). Scale bar: 2 cm (see Figure S3).

The nasal cavities form a bilaterally symmetric pair flanking the nasal septum. Figure 1 and Figure S1 show views of the lateral and medial cavity walls and of the turbinate complex within the cavity; Figure 2 lateral and medial views of the turbinate complex without the walls of the cavity; and Figure S2 lateral and medial views of each separate component of the turbinate complex. This comprises six lateral ectoturbinates, four medial endoturbinates, and the ventral concha; the dorsal concha is a rostral projection of endoturbinate I, and the middle concha part of endoturbinate II. Parts of ectoturbinates 2 and 3 project to the frontal sinus. Transverse sections (Figure 3 and Figure S3) clearly show the aptness of the term “complex.”

**Figure 4:**
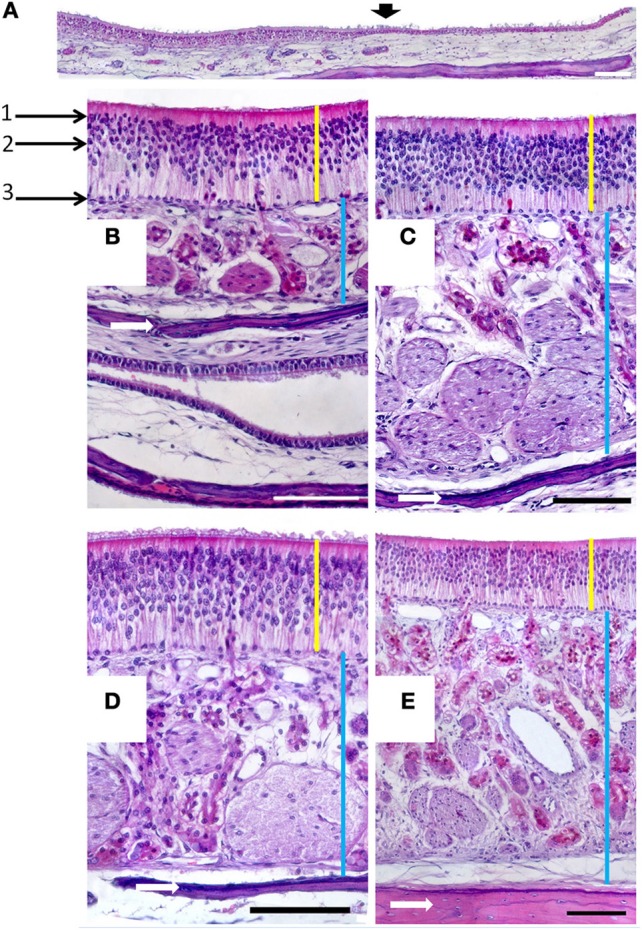
**(A)** Hematoxylin-eosin-stained longitudinal section showing the transition (arrowed) from the respiratory to the sensory epithelium. **(B–E)** Hematoxylin-eosin stained transverse sections showing the mucosa of ectoturbinate 2 **(B)**, ectoturbinate 3 **(C)**, endoturbinate III **(D)**, and the nasal septum **(E)**. Yellow bars indicate sensory epithelium; blue bars, lamina propria; and white arrows, bone. 1, supporting cells; 2, neurons; 3, basal cells. Scale bars: **(A)** 2 mm; **(B–E)** 100 μm (see Figure S4).

**Figure 5:**
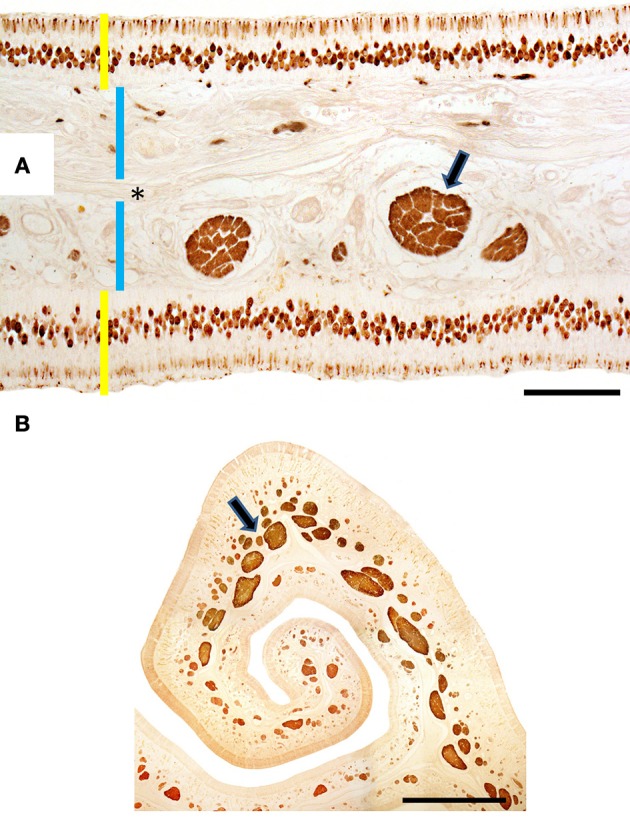
**Transverse sections of endoturbinate II stained with anti-OMP (A) and anti-G_α**o**_ (B), showing labeling of mature neurons (in A) and nerve bundles (arrowed) of the olfactory mucosa**. Yellow bars indicate sensory epithelium; blue bars, lamina propria; the asterisk, bone. Scale bars: **(A)** 100 μm; **(B)** 1 mm (see Figure S5).

In the mucosa lining the nasal cavity, color differentiates between the respiratory and olfactory epithelia, the former exhibiting shades of red or orange, the latter shades of yellow or brown (Figure 1 and Figure S1). Histological examination of selected zones of the nasal septum and of the isolated turbinates 2, 3, II, and III (Figure 4 and Figure S4) shows the transition between the two types of epithelia (Figure 4A), and also differences among different areas of sensory mucosa in regard to the thicknesses of epithelium and lamina propria and the relative thicknesses of the layers of the three epithelial cell types (sustentacular, basal, and neuronal) (Figures 4B–E and Figure S4). Immunohistochemically, the mature neurons and their apical projections of these zones were stained by anti-OMP, and the nerve bundles in the lamina propria by both anti-OMP and anti-G_α_0, but anti-G_αi_2 stained nothing specifically (Figure 5 and Figure S5). Staining consistent with these results was observed in the olfactory bulbs of dogs and mice used as controls (results not shown).

**Figure 6:**
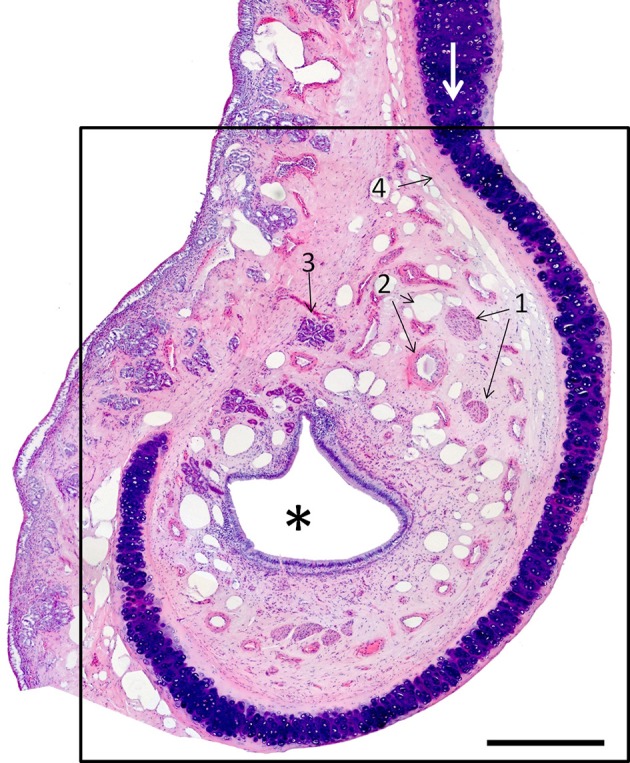
**Hematoxylin-eosin-stained transverse section of the vomeronasal organ (VNO, framed); left is lateral, up is dorsal**. The asterisk indicates the lumen of the vomeronasal duct; (1) nerves; (2) vessels; and (3) a gland. The VNO is partially surrounded by connective tissue (4) and cartilage (white arrow). Scale bar: 500 μm (see Figure S6).

The VNO, enveloped in a cartilaginous lamina, lies adjacent to the nasal septum (Figure 1). Since the VNsE is located internally, forming the central levels of the medial wall of the VNO duct, its macroscopic display would be difficult, requiring a precise, somewhat curvilinear longitudinal section through the cartilage and its content (the duct and the other soft tissues that surround it). Histologically, the VNsE is composed of basal cells, receptor neurons and supporting cells, while the anterior part of the VNO duct features stratified squamous epithelium and the posterior part simple columnar epithelium (Figure 6 and Figure S6). The immunohistochemical features of the VNsE have recently been reported elsewhere (Salazar et al., 2013) and are commented on below in the Discussion.

**Figure 7:**
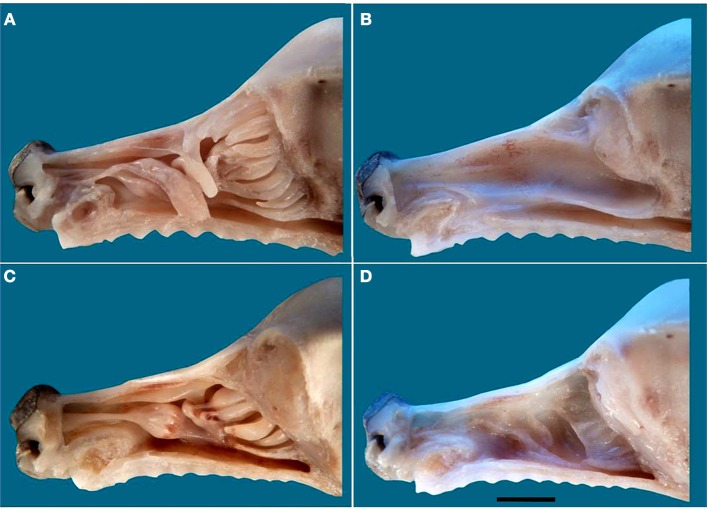
**The nasal cavity of the newborn dog**. **(A)** Lateral view of the turbinate complex. **(B)** Lateral view of the nasal septum. **(C)** Medial view of the turbinate complex. **(D)** Medial view of the lateral wall. Scale bar: 1 cm (see Figure S7).

The above descriptions all refer to adult specimens. In newborns the frontal sinus and ectoturbinate 6 are missing, and ectoturbinate 1 is very small (Figure 7). The MOE cannot usually be distinguished chromatically from respiratory epithelium, and must be identified histologically. Figure S7 shows the territory it occupies as determined by examination of series of transverse sections, which also show the simplicity and small area of the neonatal turbinates in comparison with those of adults (Figure S8).

**Figure 8:**
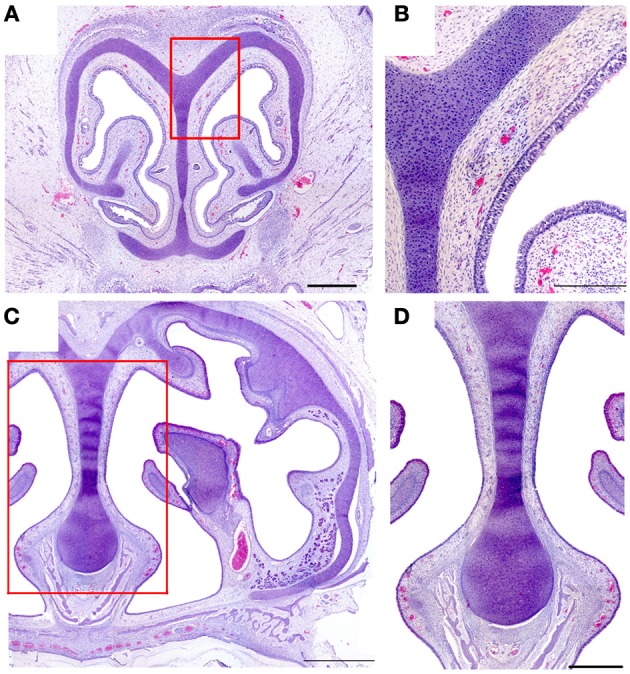
**Hematoxylin-eosin-stained transverse sections of the nasal cavity of a newborn, at the levels corresponding to the murine ganglion of Grüneberg (A) and septal organ (C)**. **(B,D)** Details of **(A,C)**, respectively. Scale bars: **(A)** 500 μm; **(B)** 250 μm; **(C)** 1000 μm; **(D)** 500 μm.

In neither adults nor newborns did we find any structure with the characteristics defining the SO and GG in mice (Barrios et al., 2014). Among our 16 fetal specimens (Figure 8 shows sections from one) there was one specimen in which the possible existence of GG cells was suggested by the presence of a nerve surrounded by vessels in the region homologous to the mouse GG, but OMP failed to label any cells in this area.

## Discussion

Among mammalian olfactory systems, the most extensively, and intensively studied has been that of the mouse—a circumstance it shares with many of the other characteristics of this animal (Paigen, 1995). It comprises four major subsystems (OSbS) stimulated via four different areas of the nasal mucosa: the MOE, the VNsE, the SO, and the GG (Barrios et al., 2014). A five-subsystem scheme emerges if the difference between vomeronasal subsystems based on semiochemical receptor types VR1 and VR2 is taken into account (Barrios et al., 2014). Although numerous studies of the canine nasal mucosa have been published (Lauruschkus, 1942; Müller, 1955; Neuhaus, 1955; Adams and Hotchkiss, 1983; Kavoi et al., 2010), we are unaware of any previous systematic search for these four or five OSbS in the dog. The findings reported above, together with our previous observation that the canine vomeronasal system binds anti-G_αi2_ but not anti-G_αo_ antibodies (Salazar et al., 2013), show that the dog has only the main and vomeronasal subsystems, and that the latter is in all probability entirely VR1-based.

Our observations of the MOE show different areas to differ considerably, especially as regards the thickness of the neuron layer and hence the surface density of neurons, even on a single turbinate. However, we were unable to organize the observed variation in a classification of mucosal types, or to relate our observations to the two-type classification proposed previously (Bock et al., 2009) on the basis of morphological characteristics, neuron turnover, and immunoreactivity.

Whereas the murine vomeronasal system expresses the VR1-associated protein G_αi2_ in the apical layer of the VNsE and the anterior half of the accessory olfactory bulb (AOB), and the VR2-associated protein G_αo_ in the basal layer of the VNsE and the posterior half of the AOB (Barrios et al., 2014), the canine system does not express G_αo_, and expresses G_αi_2 throughout the AOB as well as in the VNsE (Salazar et al., 2013). This behavior is in keeping with reported failure to find functional VR2 genes in the canine genome (Young and Trask, 2007). Given their presence in opossum (Young and Trask, 2007) and rodents (Ishii and Mombaerts, 2011), their absence from the dog (and cow and primates) (Young and Trask, 2007) must be the result of an involutionary process (Salazar and Sánchez-Quinteiro, 2009).

Regressive processes, whether evolutionary or ontogenic, seem also to have led to the absence of both SO and GG from the canine nasal cavity. Contrary to such frequently made assertions as that “the mammalian nose contains several distinct chemosensory organs, including the… MOE, the… VNO and the SO” (Tian and Ma, 2004), the SO, though present in marsupials, and in mice, rats, and rabbits, is absent from the ferret and the cat as well as the dog, (though reportedly present in fetal cat) (Breipohl et al., 1983; Kociánová et al., 2006; Ma, 2010). Similarly, statements to the effect that Grüneberg found the GG “in all mammals examined, including humans” (Tian and Ma, 2004) ignore the fact that he actually reported definitely observing it in only three of the 13 species he studied: mouse, rat, and hamster (Grüneberg, 1973). In four species (squirrel, guinea pig, shrew, and tarsier—note the two rodents) it seemed to be absent; and in the remaining six, including *Homo sapiens*, its presence was uncertain. Furthermore, he only studied two species as adults (mouse and shrew) and three more as newborns (rat, mole, and guinea-pig); all his other specimens were embryos or fetuses, and he specifically suggested that in many species the ganglion, though possibly present in these early stages, may regress during prenatal development—a process he definitely asserted of the raccoon on the basis of examination of four fetal stages.

That the dog lacks two of the olfactory epithelia of the mouse (SO and GG), and has an apparently less complex form of a third (the VNsE), may seem surprising in view of its notoriously macrosmatic nature. It may be the case that the larger size of the canine nasal cavity allows physical separation to play a larger part in the dog's discrimination among odors than is possible in the mouse (Schoenfeld and Cleland, 2005); in this respect, it must be borne in mind that our dogs were mainly German Shepherds or of German Shepherd descent, so the possibility that the SO and GG may be present in other breeds cannot be absolutely ruled out. Another possibility is that in spite of the sensitivity of the dog's sense of smell, and its ability to learn to detect unfamiliar substances by olfaction, its lifestyle prior to its domestication some 15,000 years ago (Leonard et al., 2002; Savolainen et al., 2002) required recognition of a smaller range of olfactory cues than did that of the mouse. In this regard, the dog is reported to have 811 functional olfactory receptor genes as against the 1035 of the mouse (Shi and Zhang, 2009; Niimura, 2012).

Since its domestication, the dog has of course been the object of intensive artificial selection processes that have variously pursued the enhancement of morphological, behavioral and physiological traits (regarding the mechanistic differences among these different types of modification, see Carroll, 2005, 2008; Liao et al., 2010). Creating the approximately 400 breeds of dog now estimated to exist (American Kennel Club, 2006) has altered the genome and its realization in many ways (Wayne and von Holdt, 2012), it is commonly thought that dogs collectively exhibit greater morphological variation than any other land mammal (Ostrander, 2012), and these alterations have been vigorously investigated, especially since the publication of the genome (Lindblad-Toh et al., 2005). Most of these studies have concerned the morphological traits that define breeds—body size, coat, leg length, and width, skull shape, etc. (Boyko et al., 2010; Parker et al., 2010; Schoenebeck and Ostrander, 2013), but others have addressed olfactory receptor genes, finding both marked uniformity at the gene family level, and significant variation at lower levels (Issel-Tarver and Rine, 1996; Olender et al., 2004; Tacher et al., 2005; Robin et al., 2009; Chen et al., 2012; Derrien et al., 2012; Quignon et al., 2012). This pattern suggests that our main results—the absence of SO, GG, and a VR2-based vomeronasal subsystem—seem likely to be generalizable to breeds other than German Shepherds, but confirmation is required. Light would also be thrown on this issue by a study of wolves to determine whether the absence of these subsystems is a result of domestication.

In the study of olfaction, as in other fields (Karlsson and Lindblad-Toh, 2008), the dog is proving to be a mine of information, and is leading to a much more balanced view of the mammalian olfactory system than was promoted by exclusive attention to the mouse model.

## Author contributions

Ignacio Salazar designed the research and wrote the paper. Arthur W. Barrios, and Pablo Sánchez-Quinteiro performed the work. Arthur W. Barrios, Pablo Sánchez-Quinteiro, and Ignacio Salazar analyzed and discussed the data.

### Conflict of interest statement

The authors declare that the research was conducted in the absence of any commercial or financial relationships that could be construed as a potential conflict of interest.

## References

[B1] Adams D. R. Hotchkiss D. K. (1983). The canine nasal mucosa. Anat. Histol. Embryol. 12, 109–125 10.1111/j.1439-0264.1983.tb01008.x 6225351

[B2] American Kennel Club. (2006). The Complete Dog Book, 20th Edn. New York, NY: Random House Publishing Group

[B3] Barrios W. A. Nuñez G. Sánchez-Quinteiro P. Salazar I. (2014). Anatomy, histochemistry and immunohistochemistry of the olfactory subsystems in mice. Front. Neuroanat. 8:63 10.3389/fnana.2014.00063 25071468 PMC4094888

[B4] Bock P. Rohn K. Beineke A. Baumgärtner W. Wewetzer K. (2009). Site-specific population dynamics and variable olfactory marker protein expression in the postnatal canine olfactory epithelium. J. Anat. 215, 522–535 10.1111/j.1469-7580.2009.01147.x 19788548 PMC2780570

[B5] Boyko A. R. Quignon P. Li L. Schoenebeck J. J. Degenhardt J. D. Lohmueller K. E. (2010). A simple genetic architecture underlies morphological variation in dogs. PLoS Biol. 8:e1000451 10.1371/journal.pbio.1000451 20711490 PMC2919785

[B6] Breer H. Fleischer J. Strotmann J. (2006). The sense of smell: multiple olfactory subsystems. Cell. Mol. Life Sci. 63, 1465–1475 10.1007/s00018-006-6108-5 16732429 PMC11136015

[B7] Breipohl W. Naguro T. Miragall F. (1983). Morphology of the Masera organ in NMRI mice (combined morphometric, freeze-fracture, light- and scanning electron microscopic investigations). Verh. Anat. Ges. 77, 741–743

[B8] Carroll S. B. (2005). Evolution at two levels: on genes and form. PLoS Biol. 3:e245 10.1371/journal.pbio.0030245 16000021 PMC1174822

[B9] Carroll S. B. (2008). Evo-devo and an expanding evolutionary synthesis: a genetic theory of morphological evolution. Cell 134, 25–36 10.1016/j.cell.2008.06.030 18614008

[B10] Chen R. Irwin D. M. Zhang Y-P. (2012). Differences in selection drive olfactory receptor genes in different directions in dogs and wolf. Mol. Biol. Evol. 29, 3475–3484 10.1093/molbev/mss153 22683813

[B11] Craven B. A. Neuberger T. Paterson E. G. Webb A. G. Josephson E. M. Morrison E. E. (2007). Reconstruction and morphometric analysis of the nasal airway of the dog (Canis familiaris) and implications regarding olfactory airflow. Anat. Rec. 290, 1325–1340 10.1002/ar.20592 17929289

[B12] Craven B. A. Paterson E. G. Settles G. S. (2010). The fluid dynamics of canine olfaction: unique nasal airflow patterns as an explanation of macrosmia. J. R. Soc. Interface 7, 933–943 10.1098/rsif.2009.0490 20007171 PMC2871809

[B13] Derrien T. Vaysse A. André C. Hitte C. (2012). Annotation of the domestic dog genome sequence: finding the missing genes. Mamm. Genome 23, 57–74 10.1007/s00335-011-9372-0 22076420

[B14] Graeger K. (1958). Die Nasenhöhle und die Nasennebenhöhlen beim Hund unter Besonderer Berücksichtigung der Siebbeinmuscheln. Dtsch. Tierärztl. Wschr. 65, 425–429

[B15] Granger N. Blamires H. Franklin R. J. Jeffery N. D. (2012). Autologous olfactory mucosal cell transplants in clinical spinal cord injury: a randomized double-blinded trial in a canine translational model. Brain 135, 3227–3237 10.1093/brain/aws268 23169917 PMC3501977

[B16] Graziadei P. P. Monti-Graziadei G. A. (1979). Neurogenesis and neuron regeneration in the olfactory system of mammals. I. Morphological aspects of differentiation and structural organization of the olfactory sensory neurons. J. Neurocytol. 8, 1–18 10.1007/BF01206454 438867

[B17] Grüneberg H. (1973). A ganglion probably belonging to the N. Terminalis system in the nasal mucosa of the mouse. Z. Anat. Entwicklungsgesch. 140, 39–52 10.1007/BF00520716 4749131

[B18] Ishii T. Mombaerts P. (2011). Coordinated coexpression of two vomeronasal receptor V2R genes per neuron in the mouse. Mol. Cell. Neurosci. 46, 397–408 10.1016/j.mcn.2010.11.002 21112400

[B19] Issel-Tarver L. Rine J. (1996). Organization and expression of canine olfactory receptor genes. Proc. Natl. Acad. Sci. U.S.A. 93, 10897–10902 10.1073/pnas.93.20.10897 8855279 PMC38254

[B20] Karlsson E. K. Lindblad-Toh K. (2008). Leader of the pack: gene mapping in dogs and other model organisms. Nat. Rev. Genet. 9, 713–725 10.1038/nrg2382 18714291

[B21] Kavoi B. Makanya A. Hassanali J. Carlsson H. E. Kiama S. (2010). Comparative functional structure of the olfactory mucosa in the domestic dog and sheep. Ann. Anat. 192, 329–337 10.1016/j.aanat.2010.07.004 20801626

[B22] Kociánová I. Gorošová A. Tichý F. Čížek P. Machálka M. (2006). Structure of Masera's septal olfactory organ in cat (*Felix silvestris* f. *catus)*. Light microscopy in selected stages of ontogeny. Acta Vet. Brno 75, 471–475 10.2754/avb200675040471

[B23] Lauruschkus N. (1942). Über Riechfeldgrösse und Riechfeldkoeffizient bei einigen Hunderassen und der Katze. Arch. Tierheilk. 77, 473–497

[B24] Leonard J. A. Wayne R. K. Wheeler J. Valadez R. Guillen S. Vila C. (2002). Ancient DNA evidence for old world origin of new world dogs. Science 298, 1613–1616 10.1126/science.1076980 12446908

[B25] Liao B-Y. Weng M-P. Zhang J. (2010). Contrasting genetic paths to morphological and physiological evolution. Proc. Natl. Acad. Sci. U.S.A. 107, 7353–7358 10.1073/pnas.0910339107 20368429 PMC2867737

[B26] Lindblad-Toh K. Wade C. M. Mikkelsen T. S. Karlsson E. K. Jaffe D. B. Kamal M. (2005). Genome sequence, comparative analysis and haplotype structure of the domestic dog. Nature 438, 803–819 10.1038/nature04338 16341006

[B27] Ma M. (2010). Multiple olfactory subsystems convey various sensory signals, in The Neurobiology of Olfaction, chapter 9, ed Menini A. (Boca Raton, FL: CRC Press), 1–20 21882425

[B28] Mason S. L. Maddox T. W. Lillis S. M. Blackwood L. (2013). Late presentation of canine nasal tumours in a UK referral hospital and treatment outcomes. J. Small Anim. Pract. 54, 347–353 10.1111/jsap.12083 23718867

[B29] McEntee M. C. (2004). Neoplasms of the nasal cavity, in Respiratory Disease in Dogs and Cats, ed King L. G. (Philadelphia, PA: Saunders), 293–301 10.1016/B978-0-7216-8706-3.50041-4

[B30] Müller A. (1955). Quantitative studies on olfactory epithelium of dogs. Z. Zellforsch. Mikrosk. Anat. 41, 335–350 10.1007/BF00340605 14387124

[B31] Munger S. D. Leinders-Zufall T. Zufall F. (2009). Subsystems organization of the mammalian sense of smell. Annu. Rev. Physiol. 71, 115–140 10.1146/annurev.physiol.70.113006.100608 18808328

[B32] Neuhaus W. (1955). Die Form der Riechzellen des Hundes. Naturwissenschaften 42, 374–375 10.1007/BF00629028

[B33] Niimura Y. (2012). Olfactory receptor multigene family in vertebrates: from the viewpoint of evolutionary genomics. Curr. Genomics 13, 103–114 10.2174/138920212799860706 23024602 PMC3308321

[B34] Olender T. Fuchs T. Linhart C. Shamir R. Adams M. Kalush F. (2004). The canine olfactory subgenome. Genomics 83, 361–372 10.1016/j.ygeno.2003.08.009 14962662

[B35] Ostrander E. A. (2012). Introduction [to the special issue on advances in the canine system for genetic studies]. Mamm. Genome 23, 1–2 10.1007/s00335-012-9389-z 22258618

[B36] Paigen K. (1995). A miracle enough: the power of mice. Nat. Med. 1, 215–220 10.1038/nm0395-215 7585036

[B37] Parker H. G. Shearin A. L. Ostrander E. A. (2010). Man's best friend becomes biology's best in show: genome analyses in the domestic dog. Annu. Rev. Genet. 44, 309–336 10.1146/annurev-genet-102808-115200 21047261 PMC3322674

[B38] Quignon P. Rimbault M. Robin S. Galibert F. (2012). Genetics of canine olfaction and receptor diversity. Mamm. Genome 23, 132–143 10.1007/s00335-011-9371-1 22080304

[B39] Robin S. Tacher S. Rimbault M. Vaysse A. Dréano S. André C. (2009). Genetic diversity of canine olfactory receptors. BMC Genomics 10:21 10.1186/1471-2164-10-21 19144169 PMC2635374

[B40] Salazar I. Cifuentes J. M. Sánchez-Quinteiro P. (2013). Morphological and immunohistochemical features of the vomeronasal system in dogs. Anat. Rec. 296, 146–155 10.1002/ar.22617 23161754

[B41] Salazar I. Sánchez-Quinteiro P. (2009). The risk of extrapolation in neuroanatomy: the case of the mammalian vomeronasal system. Front. Neuroanat. 3:22 10.3389/neuro.05.022.2009 19949452 PMC2782799

[B42] Savolainen P. Zhang Y. P. Luo J. Lundeberg J. Leitner T. (2002). Genetic evidence for an East Asian origin of domestic dogs. Science 298, 1610–1613 10.1126/science.1073906 12446907

[B43] Schoenebeck J. J. Ostrander E. A. (2013). The genetic of canine skull shape variation. Genetics 193, 317–325 10.1534/genetics.112.145284 23396475 PMC3567726

[B44] Schoenfeld T. A. Cleland T. A. (2005). The anatomical logic of smell. Trends Neurosci. 28, 620–627 10.1016/j.tins.2005.09.005 16182387

[B45] Shi P. Zhang J. (2009). Extraordinary diversity of chemosensory receptor gene repertoires among vertebrates. Results Probl. Cell Differ. 47, 1–23 10.1007/400_2008_4 19145414

[B46] Tacher S. Quignon P. Rimbault M. Dreano S. Andre C. Galibert F. (2005). Olfactory receptor sequence polymorphism within and between breeds of dogs. J. Hered. 96, 812–816 10.1093/jhered/esi113 16251519

[B47] Tian H. Ma M. (2004). Molecular organization of the olfactory septal organ. J. Neurosci. 24, 8383–8390 10.1523/JNEUROSCI.2222-04.2004 15385621 PMC2227317

[B48] Turek M. M. Lana S. E. (2012). Canine nasosinal tumors, in Small Animal Clinical Oncology, eds Withrow S. J. Vail D. M. (St. Louis, MO: Saunders), 525–540

[B49] Wayne R. K. von Holdt B. M. (2012). Evolutionary genomics of dog domestication. Mamm. Genome 23, 3–18 10.1007/s00335-011-9386-7 22270221

[B50] Young J. M. Trask B. J. (2007). V2R gene families degenerated in primates, dog and cow, but expanded in opossum. Trends Genet. 23, 212–215 10.1016/j.tig.2007.03.004 17382427

