# Supplementary material for: Dog and mouse: toward a balanced view of the mammalian olfactory system
Source: Front Neuroanat. 2014 Sep 25;8:106. doi: 10.3389/fnana.2014.00106 (PMC4174761; doi:10.3389/fnana.2014.00106)
Supplement: Supplementary file 1 [file Presentation1.PDF]

Frontiers in Neuroanatomy

Supplementary Material

Dog and mouse:
Towards a balanced view of the mammalian olfactory system

Arthur W. Barrios, Pablo Sánchez-Quinteiro and Ignacio Salazar*

Department of Anatomy and Animal Production, Unit of Anatomy and Embryology, Faculty of Veterinary, University of Santiago de Compostela. Lugo, Spain

*Correspondence:
Ignacio Salazar, Department of Anatomy and Animal Production, Unit of Anatomy and Embryology, Faculty of Veterinary, University of Santiago de Compostela, Av Carballo Calero s/n, 27002 Lugo, Spain.
e-mail: ignacio.salazar@usc.es

Figure S1

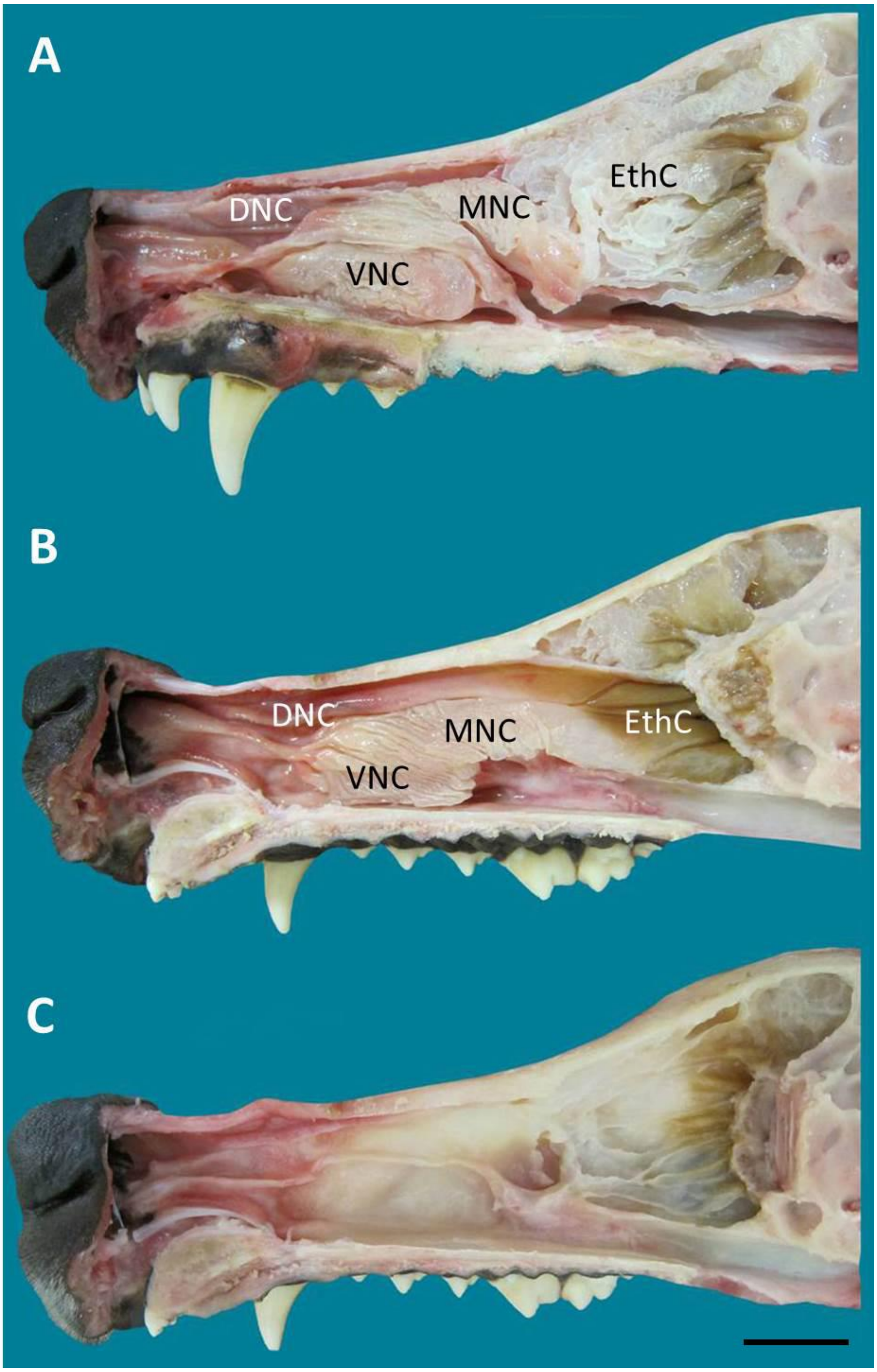

Figure S1. Lateral (A) and medial (B) views of the turbinate complex in the nasal cavity of the adult dog. C, medial view of the lateral wall of the nasal cavity. Note the difference in colour between the yellow-brown sensory mucosa and the red-orange respiratory mucosa. DNC, dorsal nasal concha; EthC, ethmoturbinate concha; MNC, middle nasal concha; VNC, ventral nasal concha. Scale bar: 2cm.

Figure S2

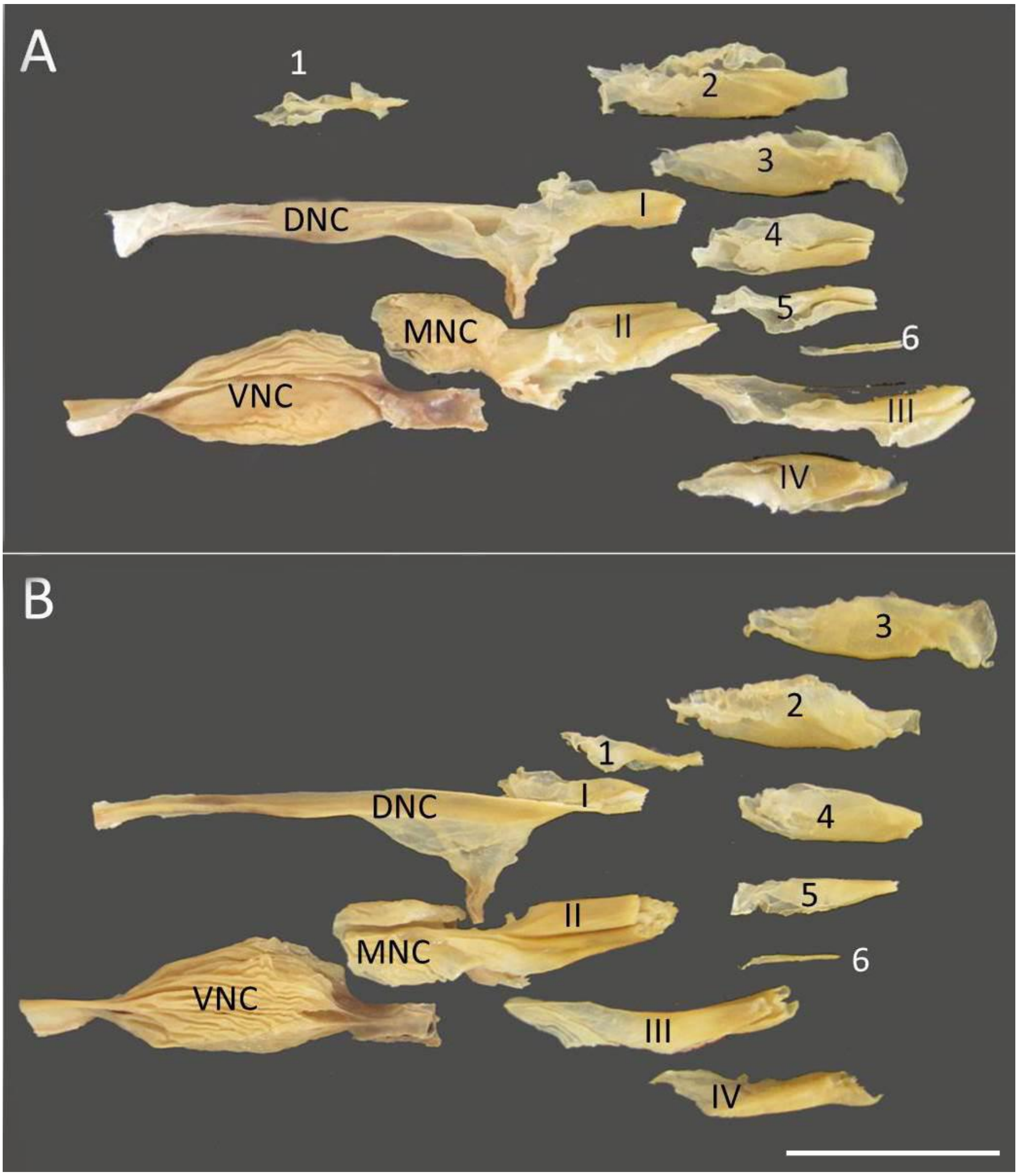

Figure S2. The isolated turbinates in lateral (A) and medial (B) views. DNC, dorsal nasal concha; MNC, middle nasal concha; VNC, ventral nasal concha. Ectoturbinates are identified by Arabic numerals (1-6) and endoturbinates by roman numerals (I-IV). Scale bar: 2cm.

Figure S3-1

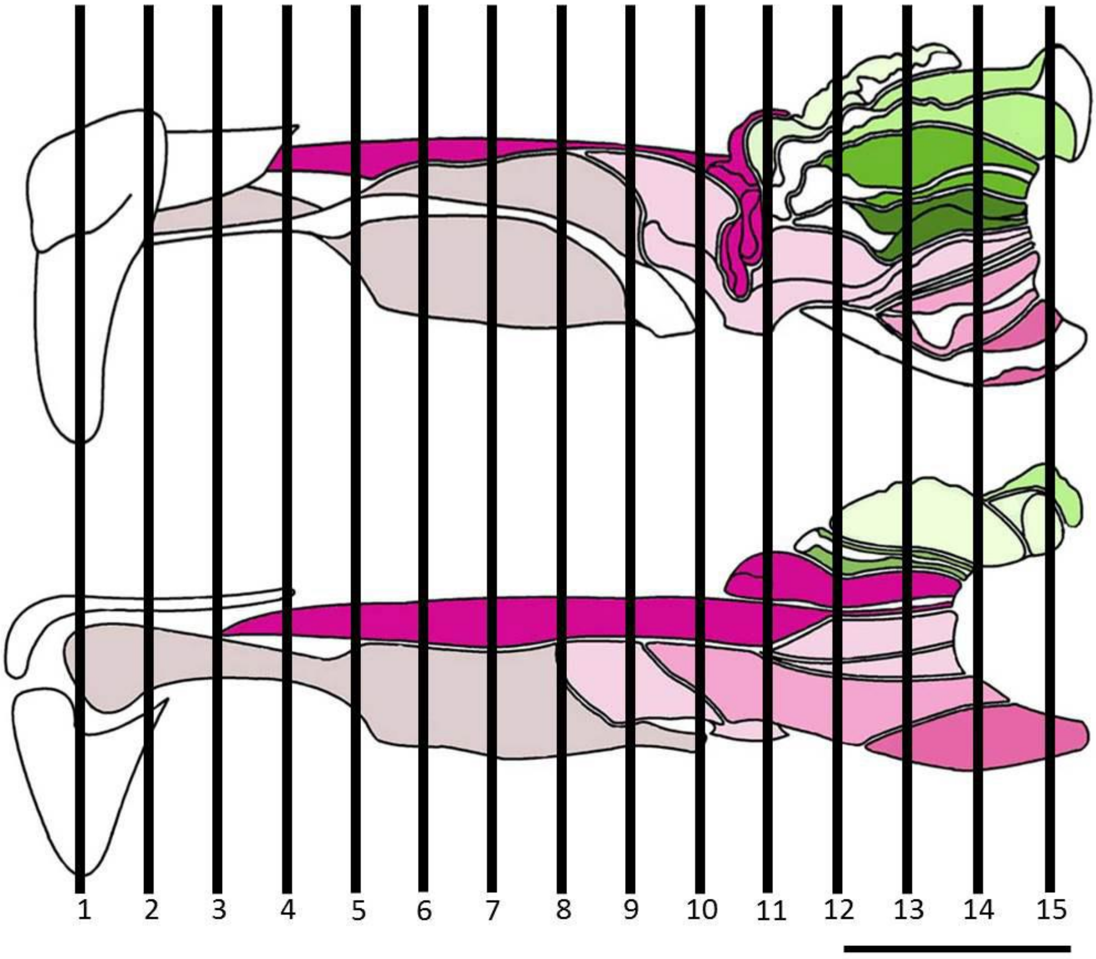

Figure S3-1. Schematic drawings of the turbinate complex of the nasal cavity in lateral (top) and medial (bottom) views, showing the levels of the transverse sections of Figure S3.2. Scale bar: 2cm.

Figure S3-2

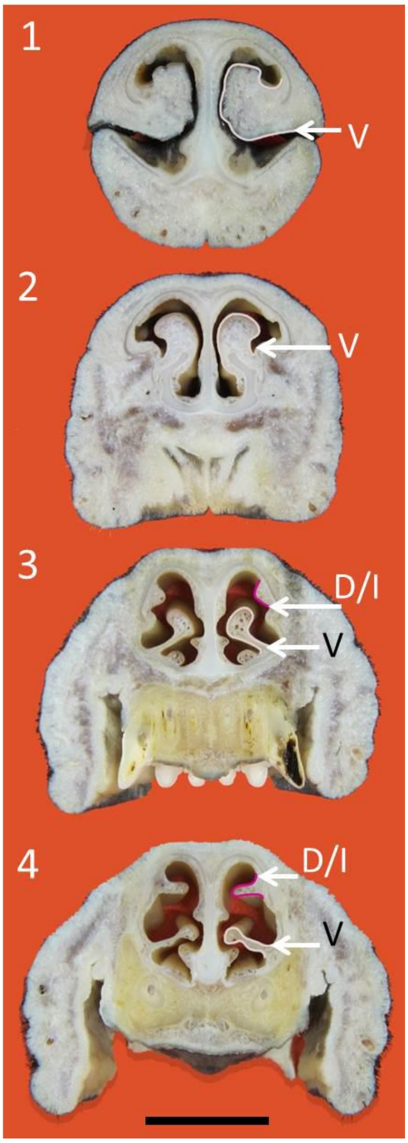

Figure S3-2. Transverse frozen sections of the nasal cavity at the levels indicated in Figure S3.1. D, dorsal nasal concha; M, middle nasal concha; V, ventral nasal concha. Ectoturbinates are identified by Arabic numerals (1-6) and endoturbinates by roman numerals (I-IV). Scale bars: 2cm.

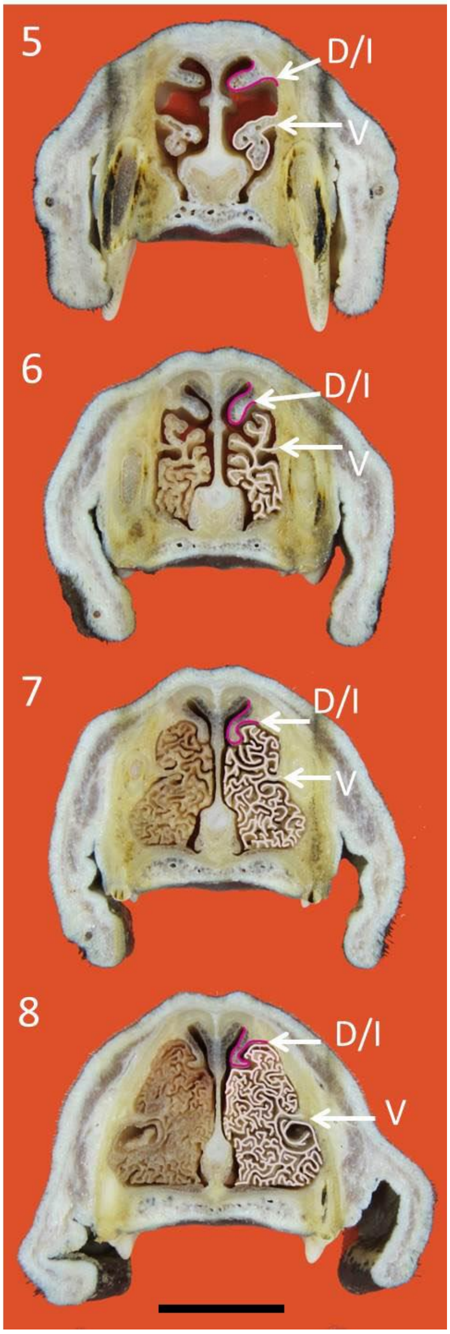

5
D/I
V
6
D/I
V
7
D/I
V
8
D/I
V

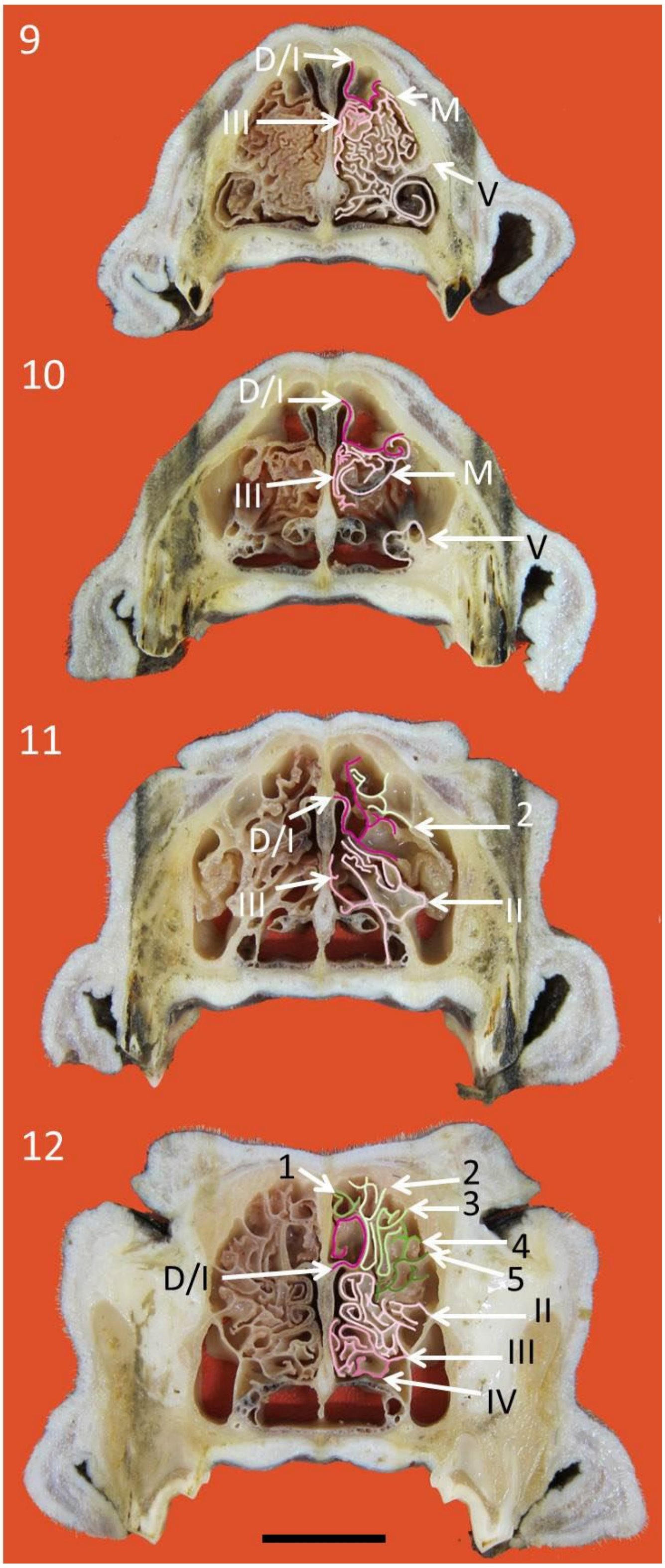
9
D/I
III
M
V
10
D/I
III
M
V
11
D/I
III
2
II
12
1
2
3
4
5
D/I
II
III
IV

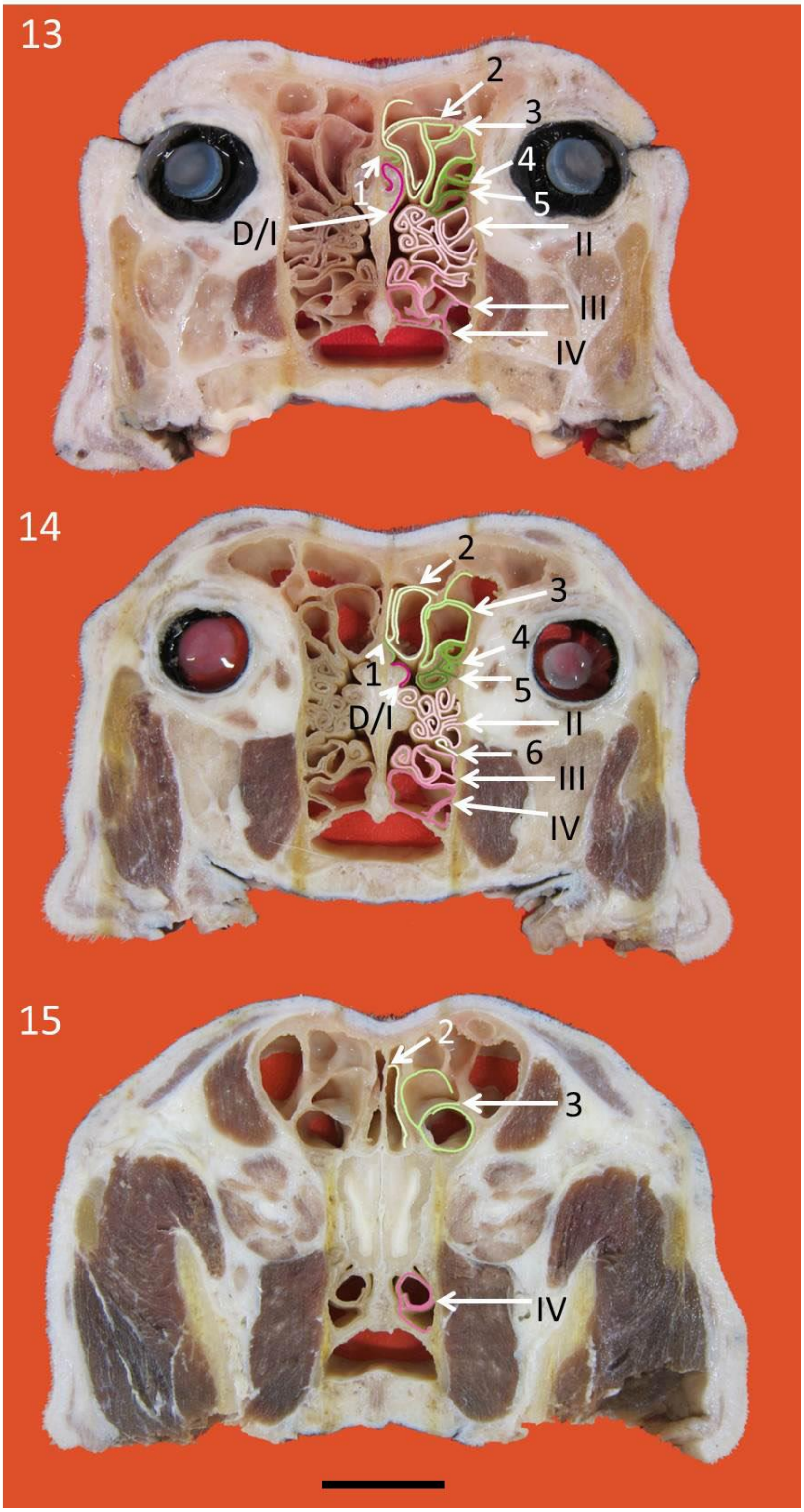
13
2
3
4
1
5
D/I
II
III
IV
14
2
3
4
1
5
D/I
II
6
III
IV
15
2
3
IV

Figure S4

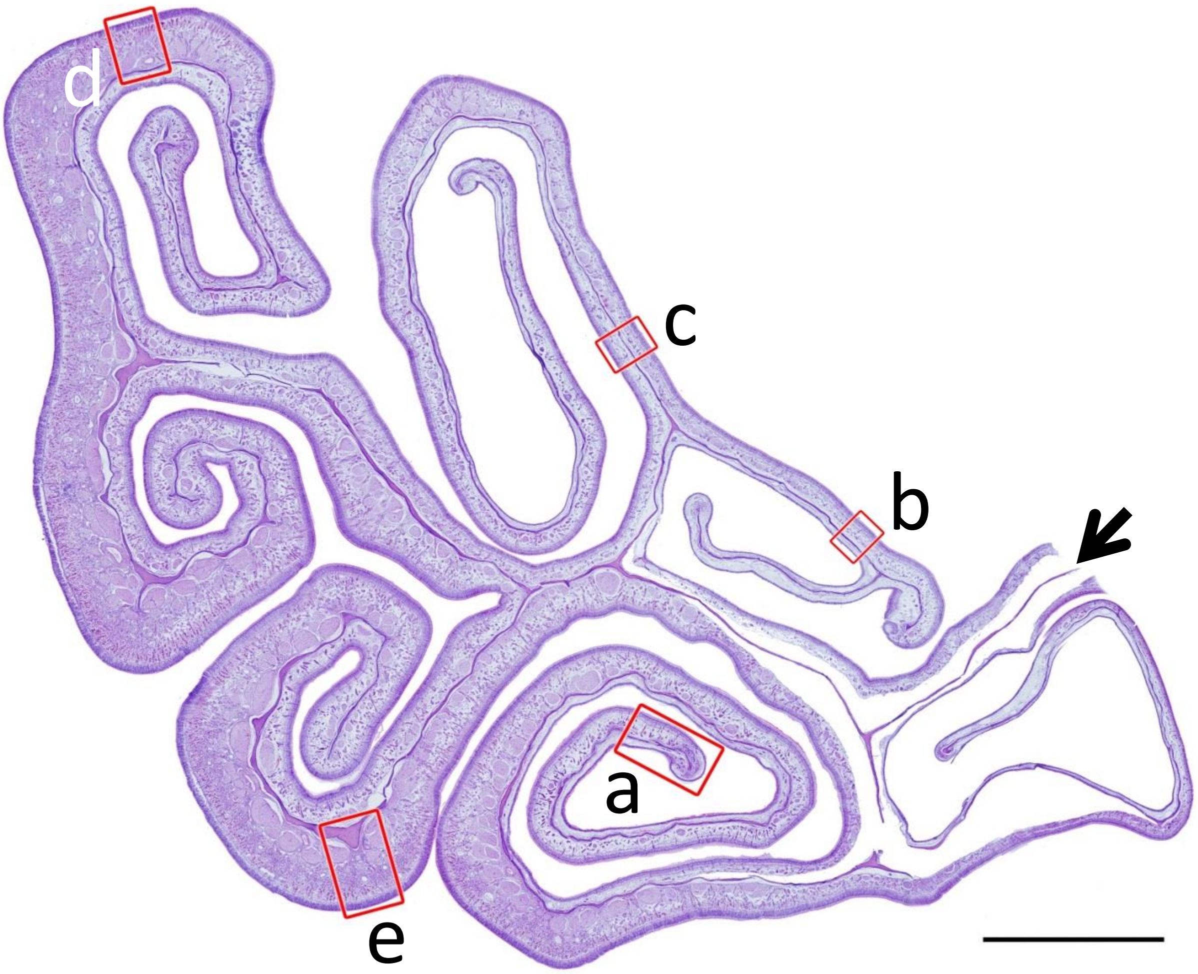

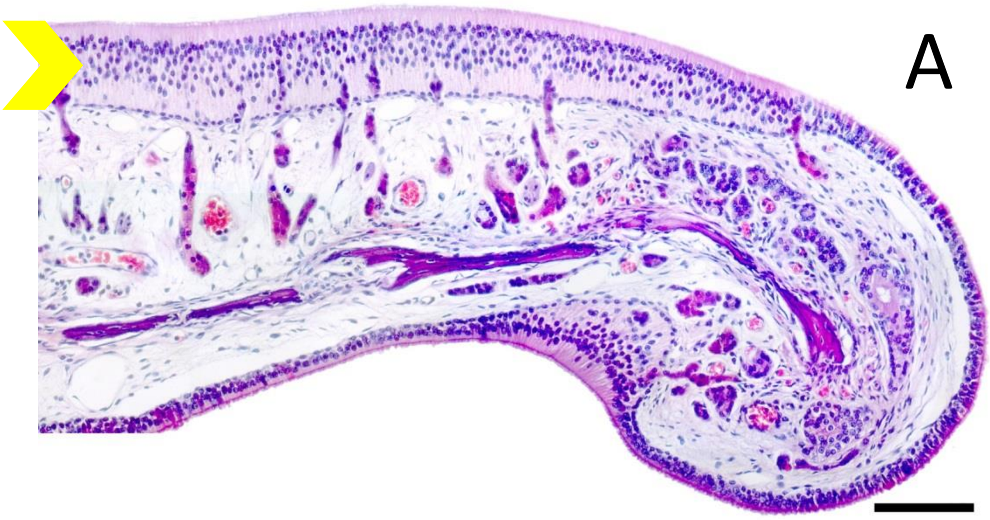

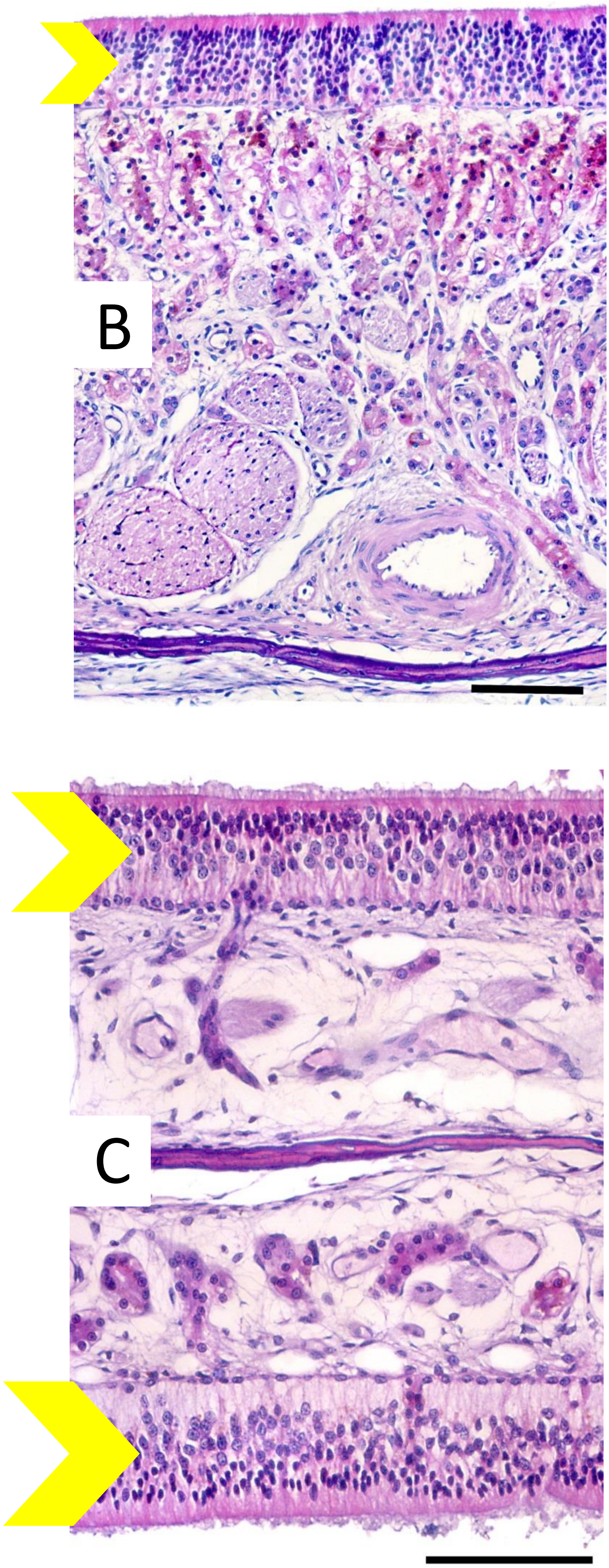
B
C

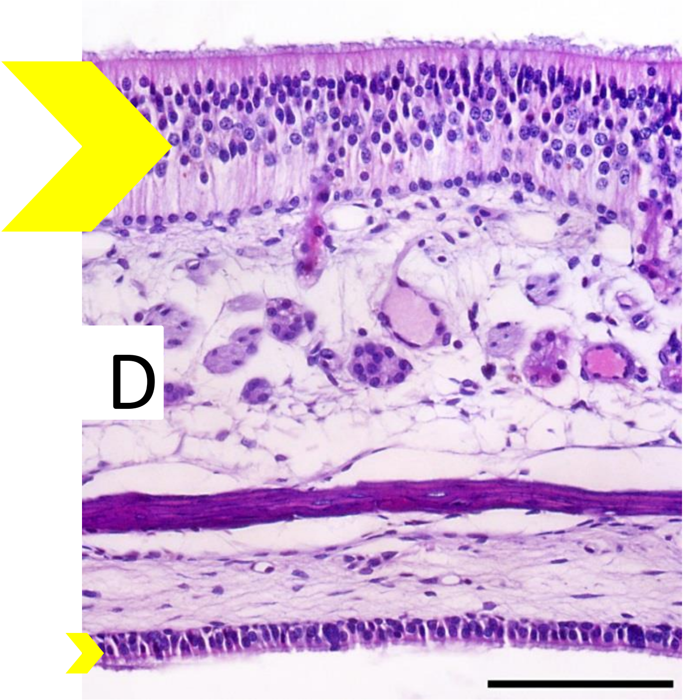

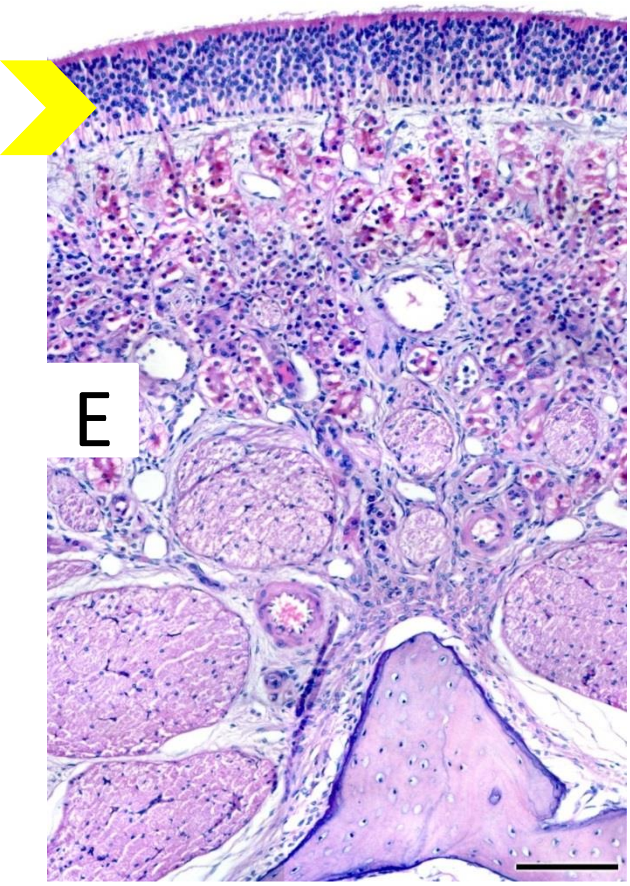

Figure S4. Haematoxylin-eosin-stained transverse section of endoturbinate II, with the osseous basal lamina arrowed (left, medial; up, dorsal). A-E: Details corresponding to boxes a-e. Note the differences between various areas of sensory epithelium (yellow arrows). Scale bars: whole endoturbinate image, 2mm; A-E, 100µm.

Figure S5

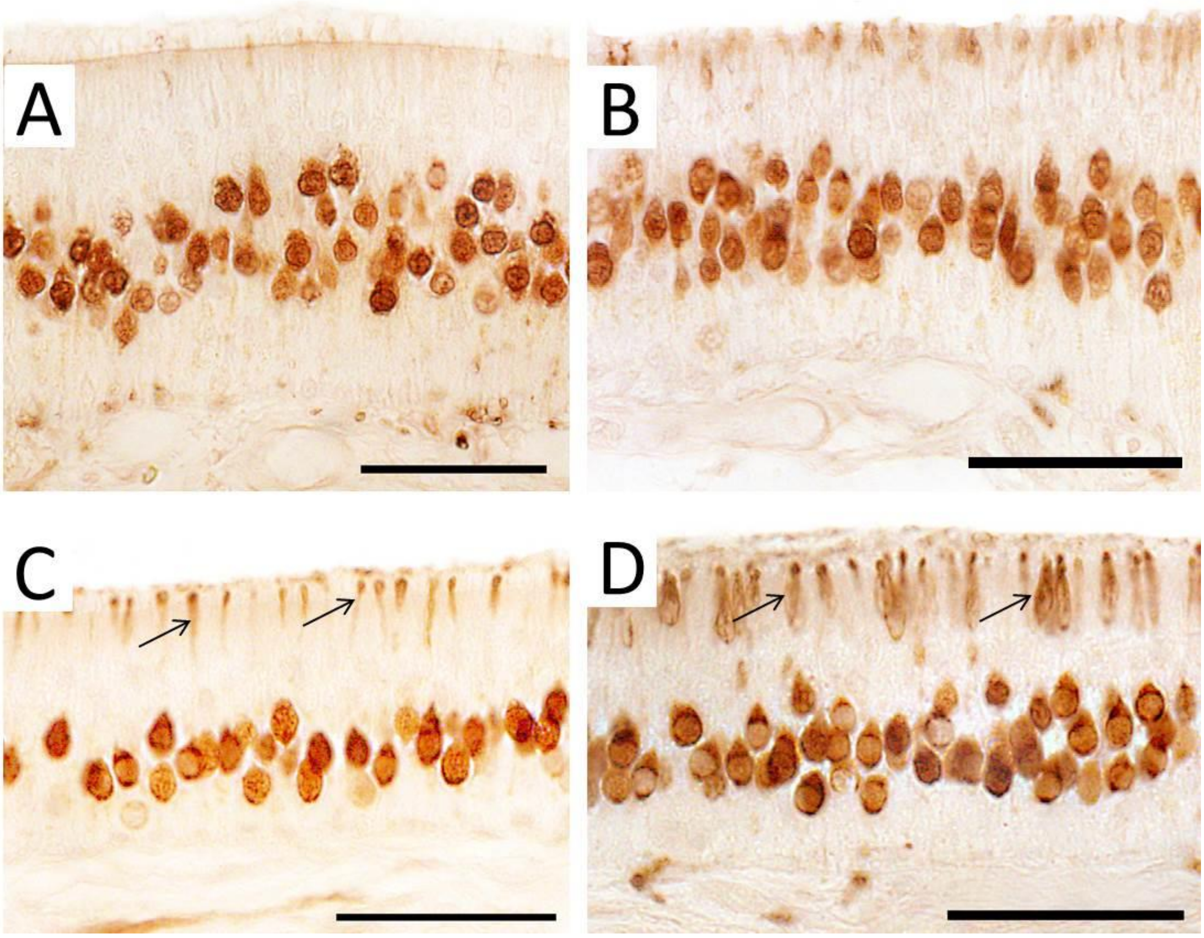

Figure S5. Transverse sections of the sensory epithelium of endoturbinate II at different levels (A, most lateral, D, most medial), showing labelling of mature neurons and their apical projections (arrowed in C and D) by anti-OMP. Scale bars: 50µm.

Figure S6-1

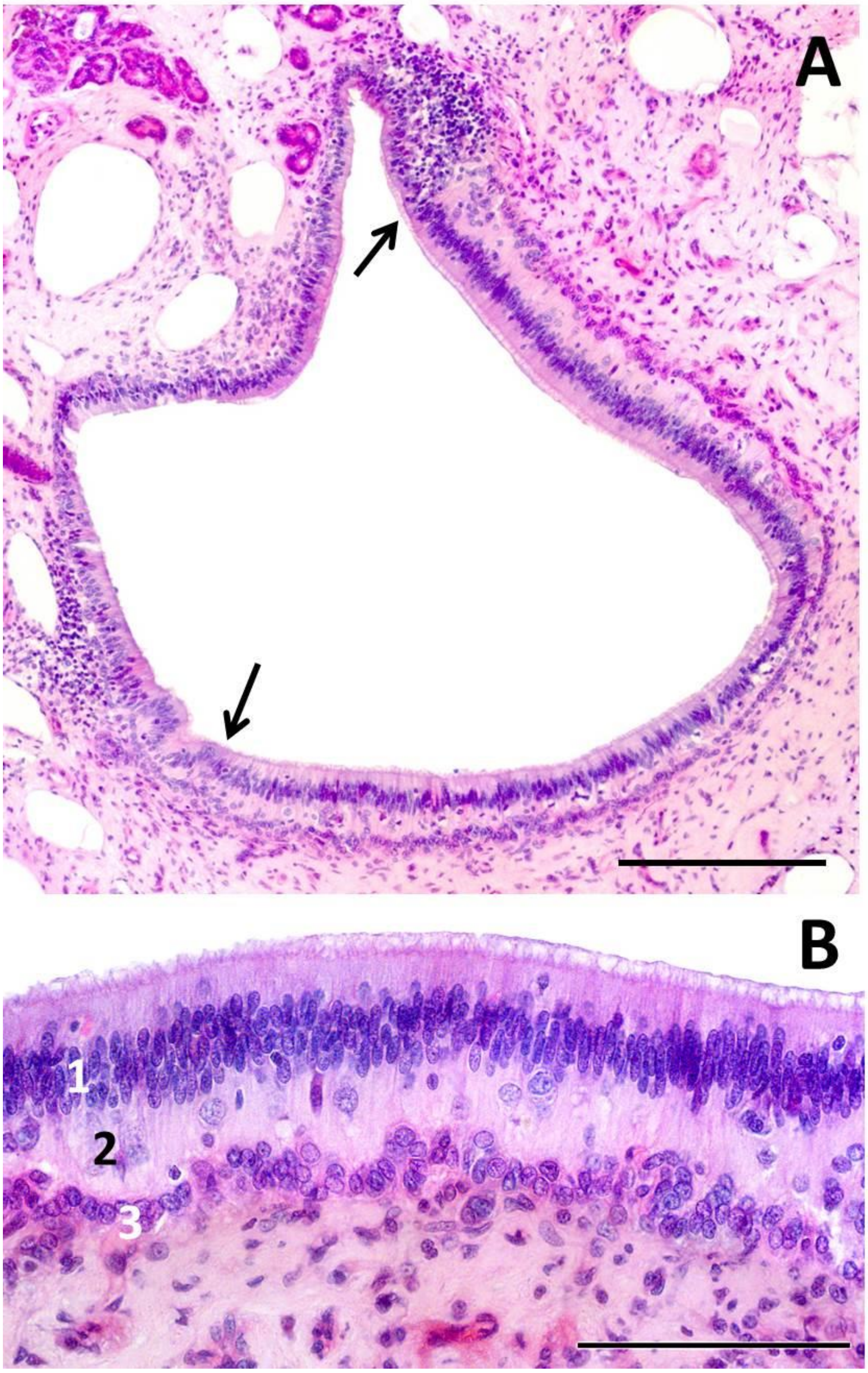

Figure S6.1. Haematoxylin-eosin-stained transverse sections of A) the central area of the vomeronasal duct (the borders of the sensory epithelium are arrowed), and B) the vomeronasal sensory epithelium. 1, supporting cells; 2, neurons; 3, basal cells. Scale bars: A, 250µm; B, 100µm.

Figure S6-2

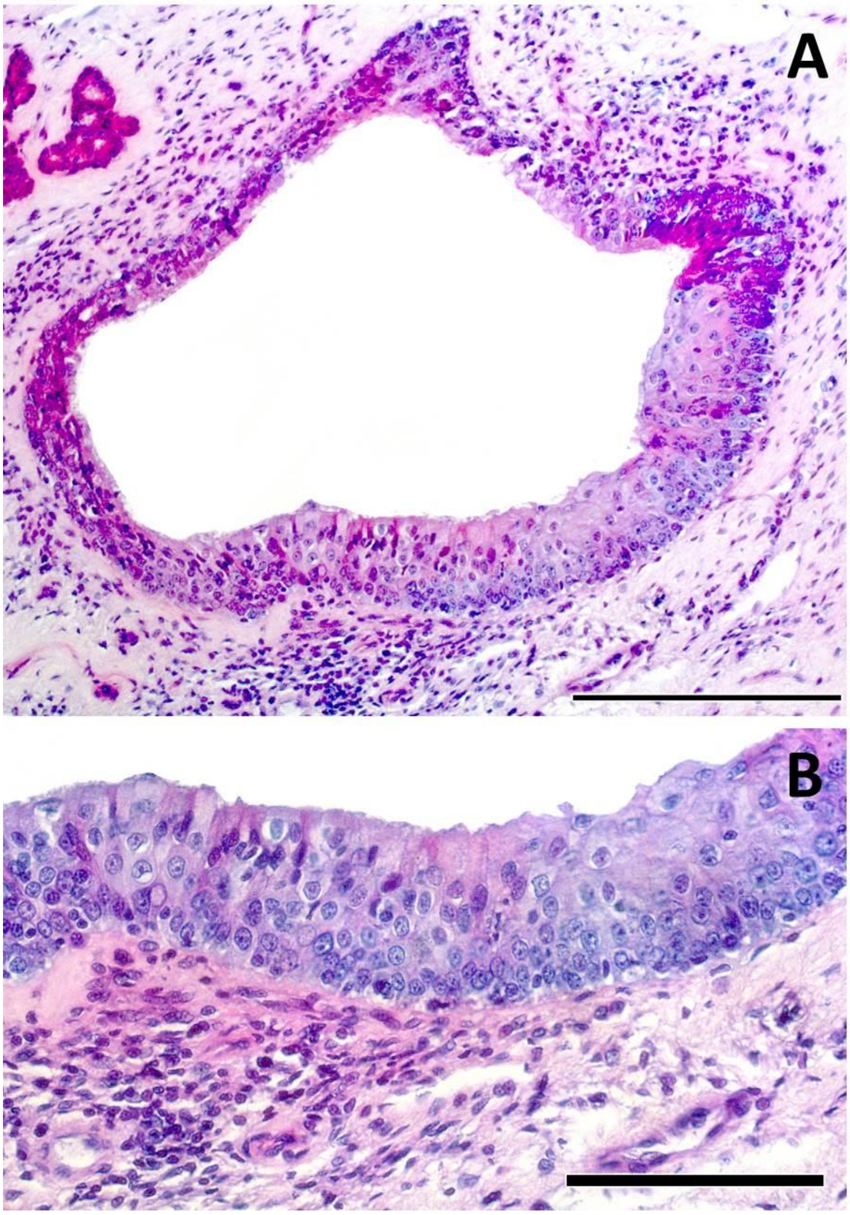

Figure S6.2. (A) Hematoxylin-eosin stains transverse section of the most anterior part of the vomeronasal duct, showing no sensory epithelium. (B) High magnification of A. Scale bars: 200µm (A), 100µm (B).

Figure S6-3

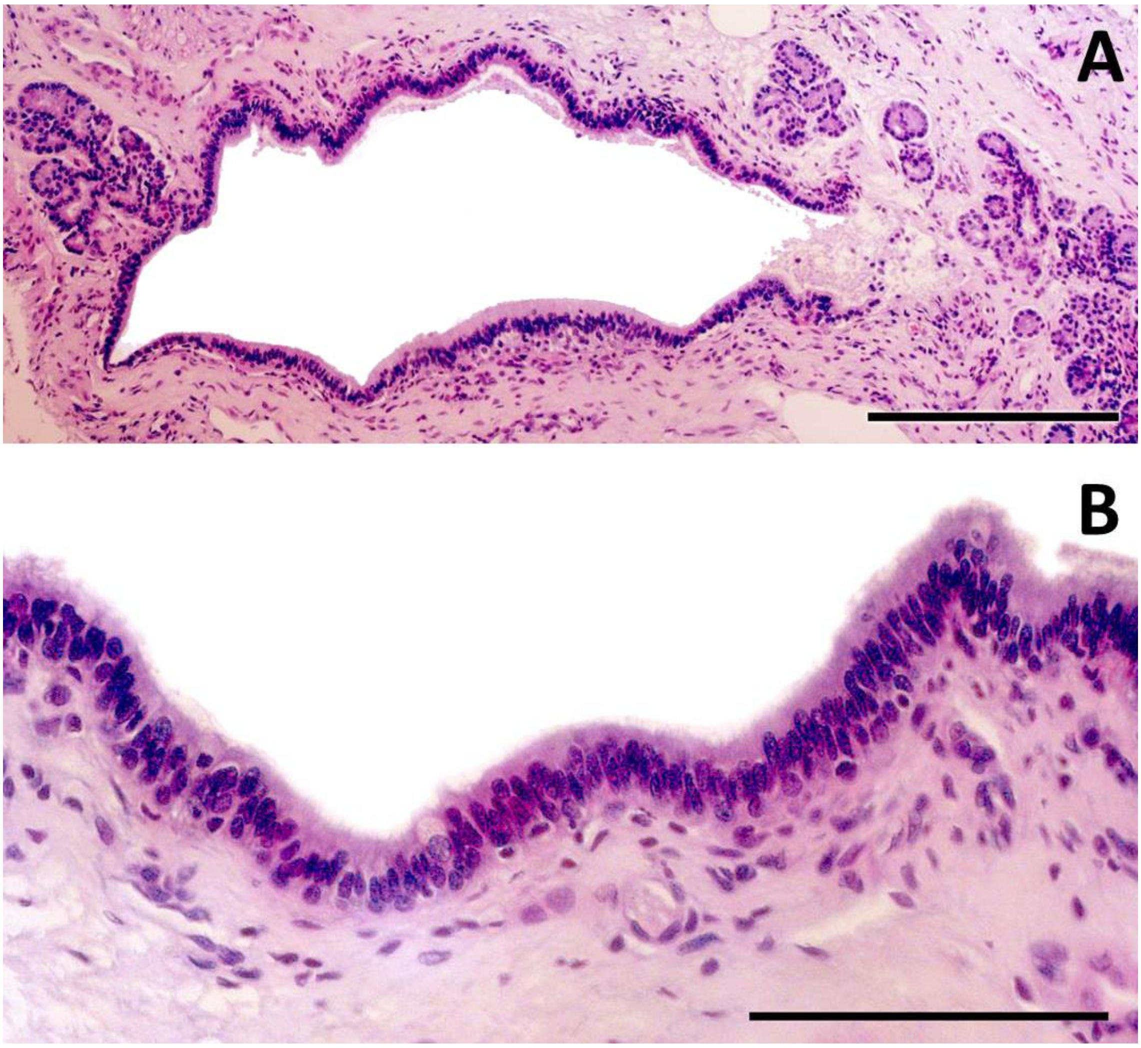

Figure S6.3. (A) Hematoxylin-eosin stains transverse section of the most posterior part of the vomeronasal duct, showing no sensory epithelium. (B) High magnification of A. Scale bars: 200μm (A), 100μm (B).

Figure S7

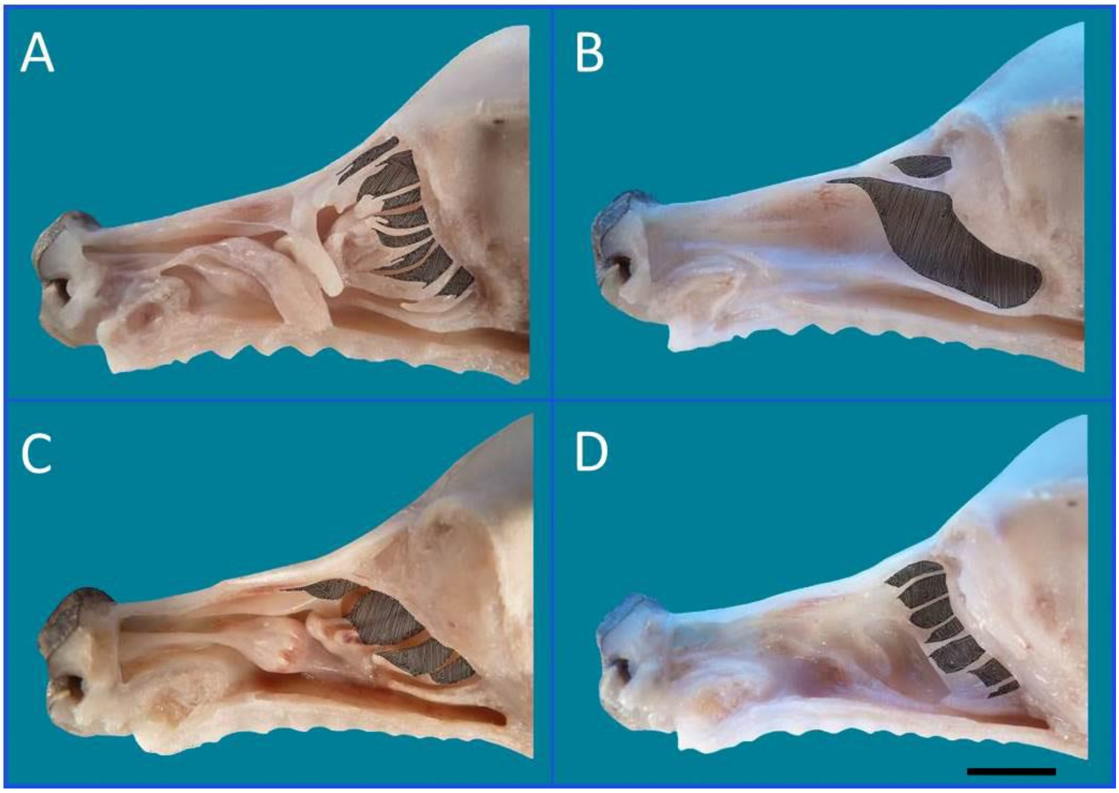

Figure S7. The nasal cavity of the newborn dog, showing the territory occupied by the sensory epithelium in artificial dark colour. A, lateral view of the turbinate complex. B, lateral view of the nasal septum. C, medial view of the turbinate complex. D, medial view of the lateral wall. Scale bar: 1cm.

Figure S8

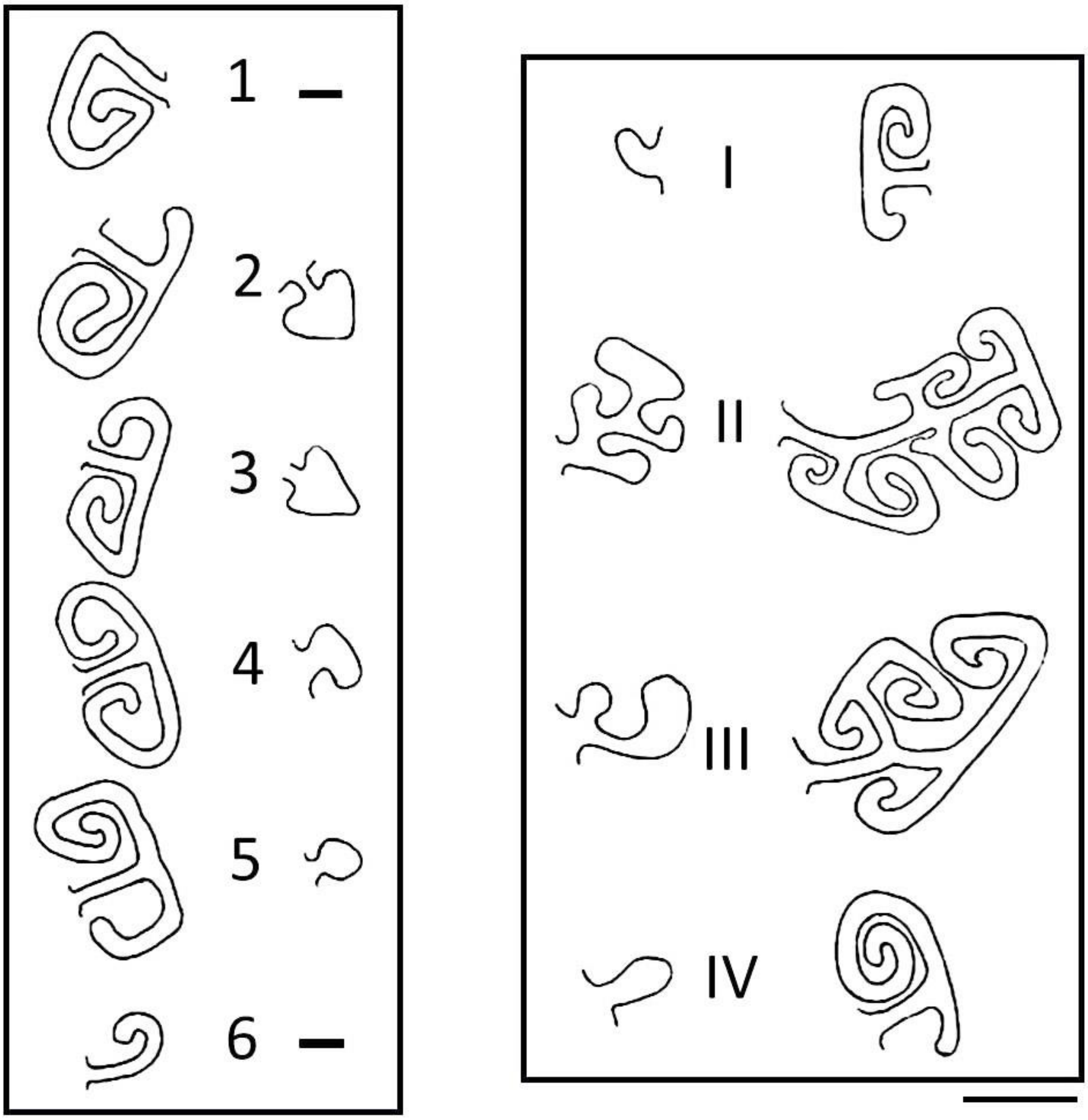

Figure S8. Schematic representation showing the different degree of development of the ectoturbinates (1-6) and endoturbinates (I-IV) in newborns (right-hand ecto- and left-hand endoturbinates) and adults (left-hand ecto- and right-hand endoturbinates). Scale bar: 5mm.
